# PPARα: An emerging target of metabolic syndrome, neurodegenerative and cardiovascular diseases

**DOI:** 10.3389/fendo.2022.1074911

**Published:** 2022-12-16

**Authors:** Yijun Lin, Yan Wang, Pei-feng Li

**Affiliations:** ^1^ Xiamen Cardiovascular Hospital of Xiamen University, School of Medicine, Xiamen University, Xiamen, China; ^2^ Xiamen Key Laboratory of Cardiovascular Disease, Xiamen, China

**Keywords:** PPARα (peroxisome proliferator-activated receptor alpha), transcription, MAFLD, diabetes, alzhaimer’s disease (AD), cardiovascular diseases, cancer

## Abstract

Peroxisome proliferator-activated receptor α (PPARα) is a ligand-activated transcription factor that is involved in lipid metabolism of various tissues. Different metabolites of fatty acids and agonists like fibrates activate PPARα for its transactivative or repressive function. PPARα is known to affect diverse human diseases, and we focus on advanced studies of its transcriptional regulation in these diseases. In MAFLD, PPARα shows a protective function with its upregulation of lipid oxidation and mitochondrial biogenesis and transcriptional repression of inflammatory genes, which is similar in Alzheimer’s disease and cardiovascular disease. Activation of PPARα also prevents the progress of diabetes complications; however, its role in diabetes and cancers remains uncertain. Some PPARα-specific agonists, such as Wy14643 and fenofibrate, have been applied in metabolic syndrome treatment, which might own potential in wider application. Future studies may further explore the functions and interventions of PPARα in cancer, diabetes, immunological diseases, and neurodegenerative disease.

## Introduction

Peroxisome proliferator-activated receptor α (PPARα) is a ligand-activated transcription factor belonging to the NR1C nuclear receptor subfamily. Together with PPARγ and PPARβ/δ, all PPARs are responsible for the metabolism of lipid and glucose, as well as cell proliferation and differentiation, inflammation, vascular biology, and cancer (1). The expression of the three PPARs is quite different between organs, indicating their distinct physiological roles (2). PPARα is highly expressed in hepatocytes, cardiomyocytes, proximal renal tubular cells, and brown adipocytes. PPARβ/δ is more ubiquitous but mainly found in skeletal muscle, skin, adipose tissue, heart, liver, and inflammatory cells, whereas the localization of PPARγ is wider.

As the first identified member of the family in 1990, PPARα was found activated by a diverse class of rodent hepatocarcinogens that causes proliferation of peroxisomes (3). Subsequently, the other two members were identified and this family was verified as transcriptional factors (4, 5). PPARs are activated by various ligands, including different metabolites of fatty acids. However, most of the ligands and agonists seem not very selective, partly resulting from the highly similar structure of this family (2).

PPARα, which was firstly found as a member of the steroid hormone receptor superfamily of ligand-activated transcription factors, is mainly involved in regulation of lipid oxidation. Because lipid and even energy homeostasis maintains the function of the whole body, PPARα is logically associated with various diseases. Meanwhile, the decrease of PPARα is also found in various diseases including MAFLD, diabetes, Alzheimer’s disease, and cardiovascular disease (6–9), emphasizing the key role of PPARα in human diseases. This review summarized the recent findings of PPARα in metabolic syndrome, Alzheimer’s disease, and cardiovascular disease and discussed unsolved questions of the role of PPARα in cancers.

## PPARα structure and transcriptional function

### Canonical structure of PPAR

The PPARα protein possesses five main functional domains embodied in a modular canonical structure, namely, the activation function-1 (AF-1), the DNA-binding domain (DBD), the hinge region, the ligand-binding domain (LBD), and activation function 2 (AF-2) (5). The N-amino terminal end harbors AF-1 that operates autonomously in a ligand-independent manner. DBD consists of two highly conserved zinc finger-like motifs that promote the receptor’s binding to the PPRE sequence of the target genes, localizing in gene regulatory regions and organized as direct repeats of two hexamer core sequences AGG(A/T)CA. The hinge region that bridges the DBD to the LBD acts as a docking site for cofactors. In the C-terminal region, the LBD is responsible for ligand specificity and contains AF-2, where the ligand-containing LDB stabilizes and facilitates the interface of AF-2 so that PPARα can recruit co-activators (10).

### PPARα-dependent transactivation

It is worthy to notice that the PPAR family, including PPARα/β/γ, binds PPREs uniquely as heterodimers with the retinoid X receptor (RXR) (11). PPRE contains two core sequences separated by one nucleotide (DR-1), which provides a polarization signal to the PPAR/RXR heterodimer, whereas PPARs interact with 5′-extended hexamers and RXR binds to the downstream motif (12). Ligand-activated PPARα also recruits numerous co-activator proteins to form the transcriptionally active PPARα-interacting cofactor complex, depending on the AF-2 domain of PPARα (13). The complex contains members of the CBP/p300 and SRC/p160 family exhibiting HAT activity and the large complex of PBP/MED1 for transcription (14).

PPARα recognizes PPRE to activate transcription; however, it is interesting that almost half of the PPARα-binding regions in human hepatocytes are located within introns, whereas only 26% was in the promoter region (<2.5 kb). In addition, overlap chromatin binding regions of LXR–RXR and PPARα–RXR and co-enrichment of PPARα-binding regions in C/EBPα and TBP motifs together suggest that PPARα may influence gene expression through the formation of complexes (15).

PPARα mainly regulates the expression of genes involved in fatty acid transport and oxidation to control lipid homeostasis. Hepatic PPARα activity controls the expression of apolipoprotein, as functional PPRE has been identified in the promoters of the LPL, APOA5, APOA1, and APOA2 genes (16), whereas PPARα regulates ABCA1 in macrophage and intestine. Fatty acid oxidation in the liver and brown adipose tissue is also affected by PPARα-mediated transactivation. The expressions of Acox1, Cpt1, and Ehhadh, three important genes in mitochondrial fatty acid β-oxidation, are directly enhanced by PPARα (15). Moreover, Fgf21, a secretory hepatic factor participating in regulation of energy balance, is also a target of PPARα (17).

### Transcriptional repression

The models of PPARα transcriptional repression include PPRE-dependent or independent patterns. The independent manner of negative regulation is *via* protein–protein interactions, where a well-known example is that PPARα represses pro-inflammatory signaling pathways in acute inflammation. There are direct physical interactions between PPARα, the p65 Rel homology domain, and the N-terminus JNK-responsive part of cJun, which reduces IL-6 gene expression through the AP-1 and NF-κB signaling pathways (18). Moreover, ligand activation of GR and PPARα leads to the enhanced repression of IL-6 transcriptional activity, by the mechanism that stems from a direct GR–PPARα physical interaction (12). Another mechanism of PPRE-independent transcriptional repression occurs in the ERR-driven mitochondrial respiration due to PPARα–SIRT1 complex competitive binding to the hexameric ERRE motif with ERRs (19).

A novel model of PPRE-dependent transcriptional regulation has also been proposed on the repression of IL-6 expression. Through the physical interaction between PPRE and p65, PPARα abolishes p65 binding to the upstream NF-κB response element on the complement C3 promoter (20). These studies support the complex pattern of PPARα-mediated translational regulation.

## Regulation of PPARα: transcription, modification, and agonists

### Transcriptional regulation of PPARα

The expression of PPARα, as an important nuclear factor in metabolic regulation, is reported under the effect of metabolites. Exposure of β cells to elevated glucose rapidly decreases PPARα gene expression (21), which is in requirement of phosphorylation of the sugar. A further study shows that AMPK, the energy sensor, activates the expression of PPARα, which is implicated in high glucose conditions (22). Despite that glucose and polyunsaturated fatty acid improve PPARα expression in different organs which have been reported (23, 24), the pattern is complex. Fatty acids show specific alterations on PPARα gene expression, whereas its mRNA expression was upregulated by SFA, MUFA, ALA, ARA, and DHA and downregulated by LNA and EPA (25). Recently, a human APP-dependent expression of PPARα in brains has also been found in AD patients, although the regulatory pattern is still unknown (8). Posttranscriptional regulation is also reported to play a role in PPARα expression, where miR20b suppresses PPARα expression by directly targeting its mRNA (26).

### Posttranslational modification of PPARα

It has been reported since 1996 that PPARα could be phosphorylated (27), and following research has shown that phosphorylation of PPARα activates its transcriptional function. Phosphorylation of two serine sites, S12/S21, correlates with increased transactivation of PPARα in hepatocytes and cardiac myocytes, potentially *via* decreased co-repressor interaction with NCoR or increased interaction with a certain co-activator, PGC1α (28). These two sites are both targeted by mitogen-activated protein kinases (MAPKs) and cyclin-dependent kinase 7 (CDK7), which is associated with reduced adipose mass and increased energy expenditure (29, 30). Two sites in the hinge region, Ser 179 and 230, are reported to be phosphorylated in the PKC-dependent pathway, also participating in PPARα transcriptional activity (31). Moreover, some studies also show that phosphorylation of PPARα is associated with protein stability (32). S73 phosphorylation, an important event mediated by glycogen synthase kinase β (GSKβ), leads to the degradation of PPARα (33). Gilbert’s syndrome, a mouse model that shows the protective effect against hepatic steatosis, might be mediated by increased PPARα protein levels due to the reduction of S73 phosphorylation (34).

Poly-ubiquitination and the proteasome pathway also mediate the degradation of PPARα. Early findings implicated the E3 ligase MDM2 in the regulation of PPARα protein stability (35). Recently, the E3 ubiquitin ligase HUWE1 has been reported to affect PPARα stability to control hepatic fatty acid oxidation (36). Two members of the progestin and adipoQ receptor (PAQR) family, PAQR3 and PAQR9, display the regulatory function on HUWE1 combination with PPARα (37). PAQR3 pulled PPARα to Golgi apparently bound by HUWE1, whereas PAQR9 competitively combined with HUWE1 to avoid PPARα degradation. Except the regulation of protein stability, another research finds that the muscle-specific ubiquitin ligase MuRF1 can modify PPARα with mono-ubiquitination, leading to the decreased activity of PPARα due to its export from the nucleus (38).

Two lysine residues of PPARα, K185 and K358, have been reported to be subjected to SUMOylation (28). The modification of both residues increases the repressive ability of PPARα through enhanced co-repressor recruitment; however, K358 SUMOylation only occurs in female livers, suggesting a role in sexual dimorphism (39). Moreover, methylation of PPARα has recently been reported in neurons, which might affect protein stability (40).

### Ligands and agonists

As reviewed above that the LBD domain recognizes and binds ligands, the AF-2 helix is tightly packed against the LBD core for PPARα activation. Crystallography identifies tyrosine 314 as the main determinant of isotype ligand specificity (41), which affects the interaction with cofactors for transcriptional regulation. The PPARα ligands are fatty acid derivatives formed during various metabolic pathways including lipolysis, lipogenesis, and FA catabolism. Liver-specific knockout of fatty acid synthase (FAS), an enzyme catalyzing the synthesis of FA, results in NASH which could be reversed by PPARα agonists, identifying products of FAS as PPARα activators (42), which further reported phospholipid as a FAS-dependent lipid intermediate PPARα ligand. Because disruption of ACOX1 results in elevated PPARα target gene expression, substrates of ACOX-1 are likely PPARα endogenous agonists (43). Moreover, ATGL-dependent hydrolysis of TG also yields lipid PPARα ligands (44). All these studies support lipids as the endogenous ligand of PPARα, which suggests the balance regulatory function of PPARα in lipid metabolism.

Several chemical agonists have also been developed, at least 30 kinds of PPARα agonists or antagonists according to MCE (https://www.medchemexpress.cn/). Fibrates, including gemfibrozil, fenofibrate, and ciprofibrate, are clinically used in the treatment of primary hypertriglyceridemia. However, it is noted that fibrates are weak PPARα agonists and their selectivity should be concerned, especially fibrates which might also activate PPARγ and δ and even other proteins like NRF2. In some studies, fenofibrate is found to interact with over 80 proteins (45). Moreover, the potency of synthetic PPARα agonists may differ between human and mouse receptors, such as EC50 = 18,000 nM of fenofibrate in mouse but 30,000 in human (46). Wy14643 is another typical agonist of PPARα reversing insulin resistance and hepatic steatosis (47), although it also has the disadvantage of fibrates. Nevertheless, some dual-PPAR agonists like fenofibrate and saroglitazar have also been shown effective in clinical treatment (48). Some potent and selective PPARα modulators (SPPARMs), such as K-877, GW9578, and elafibranor, are currently under development for the treatment of NAFLD and diabetes (49), respectively.

## PPARα in MAFLD: a key regulator of disease

NAFLD is the liver manifestation of the metabolic syndrome and includes the spectrum of liver steatosis (known as non-alcoholic fatty liver, NAFL) and steatohepatitis (known as non-alcoholic steatohepatitis, NASH) (50). With an increasing epidemic of obesity worldwide, the estimated global prevalence of NAFLD is over 25% (51). NAFLD is a consequence of caloric overload, which is commonly referred to as the hepatic manifestation of the metabolic syndrome. On the other side, NAFLD is strongly associated with several core components of metabolic syndrome including obesity, insulin resistance or T2DM, and dyslipidemia (52). Considering the pathogenesis and multiplicate clinical indications, metabolic dysfunction-associated fatty liver disease (MAFLD) has recently been suggested as a more appropriate overarching term (53, 54). For a more accurate description, in the following part we would use the term “MAFLD.”

**Figure 1:**
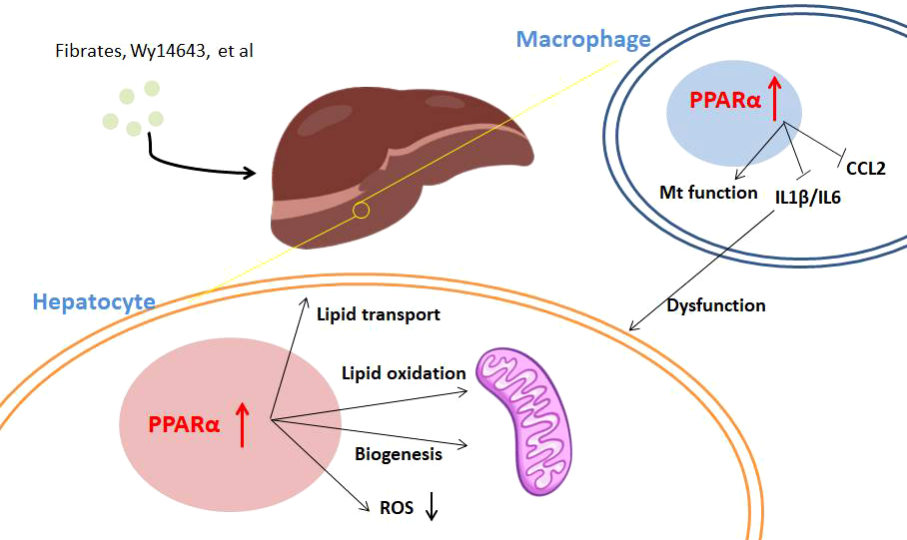
The regulation of PPARα in MAFLD. Activation of PPARα with agonists including fibrates and Wy14643 influences hepatocytes and immune cells in the liver. In hepatocytes, PPARα agonism promotes lipid transport and oxidation for lipid clearance; moreover, PPARα activation leads to ROS reduction. PPARα agonists inhibit proinflammatory cytokine production of immune cells in the liver, which protects against dysfunction of hepatocytes.

As PPARα regulates lipid metabolism and the main ligands for PPARα are fatty acids, the function of this transcriptional factor in lipid accumulation was noticed early. In the fasting state, increased fatty acid oxidation produces acetyl-CoA and promotes ketone body biogenesis, which is upregulated by PPARα (37). PPARα-deficient mice display impaired fatty acid oxidation, lipid accumulation in the liver, and an inability to augment ketone body synthesis during fasting, which indicates that PPARα is critically involved in the fasting state. Furthermore, transcriptional analysis shows that PPARα in the liver regulates fatty acid transport, peroxisomal and mitochondrial β-oxidation, and lipolysis and influences the production of apolipoproteins (55), which suggests its key role in MAFLD (**Figure 1**). Clinical data showed that liver PPARα expression inversely correlates with NASH severity, and importantly, histological improvement is associated with an increase in expression of PPARα and its target genes (6). Mouse models are in line with these experimental findings, indicating that whole-body or hepatocyte-specific deletion of PPARα promotes MAFLD in the context of obesity (56). Moreover, in preclinical models, pharmacological activation of PPARα has preventive and curative effects on NASH due to activation of hepatic transport, oxidation, and metabolism of lipids (16, 57). Mitochondrial function is also impaired in the livers of patients with NASH, who have increased hepatic oxidative stress (58). PPARα protects the liver from ROS overload *via* hydrogen peroxide detoxification and decreases hepatic ROS pools by upregulating catalase expression (16). Activation of PPARα expression by KLF16 could improve steatohepatitis and insulin resistance through ROS reduction (59). Moreover, some compounds for MAFLD, such as geniposide and fenofibrates, have recently been reported to elevate ROS levels through activating PPARα expression (60). Some studies investigate that the expression of PPARα affects fibrosis by the collagen-associated pathway. PPARα regulates NASH-related fibrogenesis through dermatopontin, which is a protein involved in fibrogenesis and collagen deposition, and its expression is lowered by PPARα activation.

Despite hepatocytes, immune cells take part in MAFLD progress especially fibrosis. On the one hand, immune activation affects the PPARα signaling pathway. Activation of the JNK pathway, which drives HFD-induced insulin resistance, increases the expression of Ncor1 and Nrip1 co-repressors to negatively regulate PPARα target expression (61). In accordance with this theory, hepatic JNK deficiency in HFD-fed mice leads to increased expression of Fgf21 for improving systemic metabolism. On the other hand, different from transactivation of lipid metabolism in hepatocytes, PPARα shows repressive function in the expression of immune genes, as discussed above in PPARα transcriptional repression. PPARα agonism resulted in reduced numbers of activated macrophages, decreased levels of IL-1β and IL-6, and improved histological evidence of liver dysfunction and endothelial function (13, 62). Pan-PPAR agonists may counteract inflammation and NASH disease progression potently (63). Moreover, PPARα agonist Wy14643 treatment could alleviate steatosis and injury of the liver and decrease the level of chemokine CCL2 (64). However, clinical assessment of the effect of PPARα selective agonists on NASH and fibrosis is still lacking.

Interestingly, as we discussed above that PPARα agonists might activate other targets, some investigators think that these targets are also beneficial to MAFLD treatment. Transcriptional factors like PPARγ and SREBP-1c are also found to be modulated by fenofibrate (65). In some ways, combining different targets, like PPAR-γ and PPAR-α combined agonist therapy, is thought to be effective in controlling fructose-induced NASH (66) and pan-PPAR agonists are found to improve in MAFLD treatment (48, 63). However, the selective and pan agonists show different functions in some research and their availability and security need further control study.

The mechanism that PPARα decreases as NASH progresses remains to lead to different theories. Although the JNK pathway has been shown to decrease PPARα expression, the regulation in the early stage of NASH might be complicated. Epigenetic mechanisms such as H3K9me3 and H3K4me3 signatures being altered in the mouse hepatic PPARα promoter might be involved in this downregulation in the model of NASH (67). Posttranscriptional silencing of PPARα is also reported to occur in hepatocytes, *via* miR10b or miR21, whose expression is enhanced during NASH (16).

## PPARα in diabetes: an effective target for diabetic complications

PPARα has been known to affect progress of type 2 diabetes. Some polymorphisms such as L162V and A268V were focused in diabetic patients, whereas L162V was early found to be associated with diabetes (68, 69). However, following genetic research showed that this mutation is associated with body mass index in patients with non-insulin-dependent diabetes mellitus (70) and fasting serum cholesterol concentrations (71), not directly affecting diabetes. Nevertheless, PPARα agonists show potential in diabetes treatment. PPARα agonists, mainly fenofibrate and Wy14643, improve glucose homeostasis by enhancing insulin sensitivity in adipose tissue and muscle (62, 72), which is probably the result of decreased lipid content in tissues by improving fatty acid β-oxidation. In addition, PPARα agonists can preserve pancreatic β-cell function, indicating that PPARα influences glucose homeostasis in part *via* effects on pancreas function (73). However, PPARα knockout also shows protective function on insulin resistance whereas fibrates do not seem to improve glucose homeostasis in humans (74, 75), making it confusing what role PPARα plays in glucose homeostasis.

**Figure 2:**
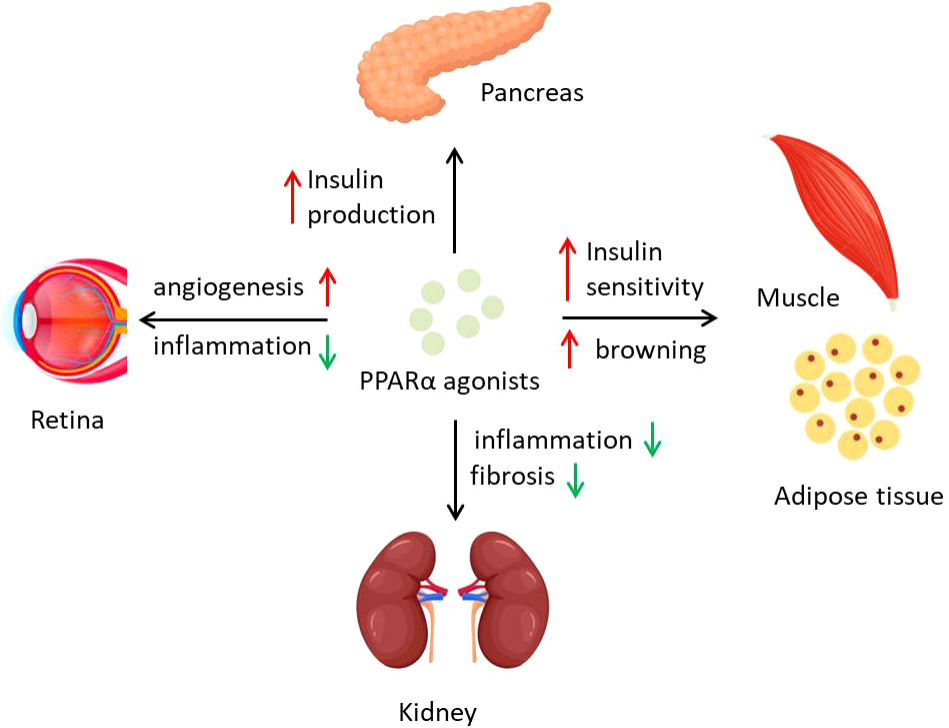
PPARα activation regulates diabetes and diabetic complications. PPARα agonists affect different organs to counter diabetes and its complications. PPARα activation preserves islets function of insulin production and promotes energy expenditure of adipose tissue and insulin sensitivity of muscle. Inflammation in kidney and retina could be inhibited by PPARα agonists, which prevent the process of DN and DR.

It is worth noting that browning of white adipocytes is thought to own the capability to counteract diabetes, which promotes lipid oxidation and glucose metabolism of the whole body (76, 77). Some early research showed that agonists for PPARα and PPARγ both affect brown adipocyte function, whereas 16 shared PPARα/γ target genes, like Ctsz, were found to regulate brown adipocyte thermogenesis (78). Activation of PPARα is reported to increase energy expenditure and insulin sensitivity in obese mice (79); meanwhile, fenofibrate is also responsible for countering brown adipose tissue whitening (80). In this aspect, PPARα activation is beneficial to glucose homeostasis through control of obesity (**Figure 2**).

Complex diabetes complications lead to poor prognosis of diabetic patients and even high risk of death. Some complications that resulted from hyperglycemia, including atherosclerosis, retinopathy, and diabetic nephropathy, are associated with PPARα function. Some studies point that PPARα expression is downregulated during diabetes, mediated by not only transcriptional regulation but also posttranslational modification (9, 40). Early research reported that naturally occurring variations of PPARα function influenced plasma lipid concentrations in type II diabetic patients but not healthy people, demonstrating that PPARα is a link between diabetes and dyslipidemia (68). Activation of PPARα by berberine is thought to attenuate diabetic atherosclerosis in ApoE^-/-^ mice (81). Fenofibrate as a PPARα agonist is used in various diabetic complications. A clinical research, field study showed that treatment with fenofibrate in individuals with type 2 diabetes mellitus reduces the need for laser treatment for diabetic retinopathy (DR) by 37%, although its mechanism might not be related to lipid oxidation but to angiogenesis (82). Following research showed that endothelial colony-forming cells (ECFCs) from PPARα^−/−^ mice displayed impaired proliferation and migration, whereas activation of PPARα by fenofibrate normalized retinal vascular degeneration (9). Another study on type 1 diabetes indicated that intraocular injection of fenofibrate ameliorates retinal inflammation in OIR rats, and these therapeutic effects on DR are PPARα dependent, suggesting PPARα as a potential target of DR cure (83). The mechanisms on why PPARα activation could reverse the DR might be partly explained by promoter methylation and protein degradation in high glucose conditions (40). PPARα has also been recently found to play roles in progress of diabetic nephropathy (DN). Lipid accumulation and metabolism are tightly associated with DN progress (84), when PPARα deficiency appears to aggravate the severity of DN through an increase in extracellular matrix formation and inflammation (85). PPARα alleviating DN seems to be mostly through alterations of inflammation, like adiponectin exerting renoprotective effects against DN by activating AMPK-PPARα (86). In addition, Annexin A1 in diabetic mice regulates the AMPK-PPARα-CPT1 pathway to attenuate inflammation in the pathogenesis of DN (87), whereas fenofibrate could also attenuate renal fibrosis through blocking the canonic Wnt signaling and activating the antioxidant effects (88), together indicating the important role of PPARα in DN.

## PPARα in Alzheimer’s disease: a potential target

Recently, there has been growing concern for the function of PPARα in the brain. PPARα protein was observed to localize in different regions of the hippocampus including CA1, CA2, CA3, and dentate gyrus (89), suggesting its role in neurodegenerative disorders. Alzheimer’s disease (AD) is one of progressive neurodegenerative diseases with classic memory impairment and cognitive disorder, where genomic locus-encoding proteins for lipid metabolism showed involvement in disease regulation (90). Some early research reported an association of the PPARα L162V polymorphism with AD risk (91), whereas recently the expression and transcriptional activity of PPARα have been found to correlate with the expression of hAPP (8), which is thought to be one of the main causes of AD. On the other side, diverse activation of PPARα has been reported to weaken AD progress. A combination of low-dose gemfibrozil and retinoic acid could induce lysosomal biogenesis through the PPARα pathway and enhance the uptake of Aβ in astrocytes to alleviate AD (92). Amyloid pathology, memory deficits, and anxiety were reversed in the mouse model of AD treated with either gemfibrozil or Wy14643, mediated by a PPARα-dependent enhancement of autophagosome biogenesis (93). Fenofibrate-mediated PPARα activation also reduces amyloidogenic processing of APP in APP/PS1 transgenic mice (94).

**Figure 3:**
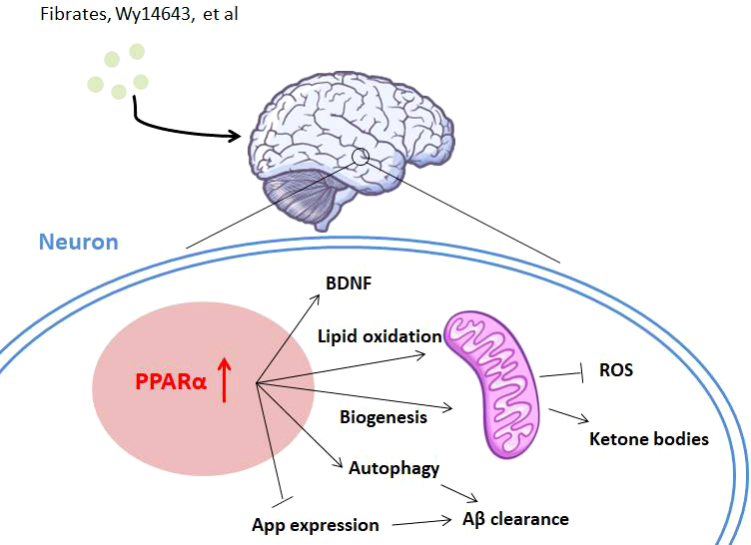
The role of PPARα in Alzheimer’s disease. PPARα is involved in Alzheimer’s disease with diverse mechanisms. Activation of PPARα promotes lipid oxidation and ketogenesis through mitochondrial biogenesis. PPARα activation also leads to Aβ clearance by App repression and autophagy of neurons. Neuroprotective factors like BDNF might also be activated by PPARα agonists for AD treatment.

Diverse mechanisms have been brought to address PPARα’s function in AD (**Figure 3**). Several studies report that PPARα plays an essential role in maintaining brain energy supply by modulating ketogenesis (90), due to ketone bodies’ function of protecting hippocampal neurons from Aβ toxicity (95, 96). Because mitochondrial disturbances play a crucial role both in aging and in neurodegenerative disorders, the regulation of PPARα in AD is also thought to be associated with mitochondrial function. In this area, some authors show that PPARα promotes glutamate transporter-1 endocytosis in astrocytes (97) and PARP1–PPARα–PGC1α regulates mitochondrial biogenesis and oxidative stress in neurons (98). Regulation of amyloid metabolism is thought to be another main function of PPARα as PPARα activity is associated with APP expression. The GW6471 PPARα antagonist inhibits APP knockdown-induced increases of synaptic activity in cortical cultures, whereas Wy14643 shows a reverse function, through the regulation of synaptic Aβ activity (8). Gemfibrozil or Wy14643 enhances autophagy in the APP-PSEN1ΔE9 mouse model to clear Aβ (93), and lysosome-mediated Aβ clearance regulated by PPARα also supports this pathway (92). Another point showed that activation of PPARα stimulates ADAM10-mediated proteolysis of Aβ in hippocampal culture (99), together indicating the regulation of Aβ metabolism by PPARα. Other pathways, like enhancing the level of brain-derived neurotrophic factor (BDNF) in the hippocampus, might partly take part in this therapy (100).

The preferential activation of PPARα also affects other neurodegenerative disorders. Fenofibrate reduces neuroinflammation and blocks neurodegeneration in a mouse model of ALS, whose mRNA analysis indicated a significant effect of this drug on transcription of anti-inflammatory and antioxidative genes (101). In another experimental animal model of Parkinson’s disease (PD), a neuroprotective effect of fenofibrate was also observed (102). Studies showing that PPARα directly participates in these diseases are still not sufficient. However, it is clear that mitochondrial metabolism, which is mediated by PPARα, plays a central role in the disorders. Whether PPARα agonists are effective for diverse neuron disorders deserves further exploration.

## PPARα in cardiovascular disease: protective effect

Cardiovascular disease (CVD) still remains the leading cause of death globally, accounting for 17.9 million deaths per year according to WHO (https://www.who.int/en/news-room/fact-sheets/detail/cardiovascular-diseases-(cvds)). Both obesity and diabetes have been implicated as major risk factors for CVD (7), suggesting the importance of lipid and glucose metabolic homeostasis in CVD. As we have discussed above on the function of PPARα in metabolic syndrome, PPARα is probably involved in the regulation of CVD. Moreover, PPARα is expressed in the vasculature and its expression is detected in ECs, vascular smooth muscle cells (VSMCs), and monocytes/macrophages, which are all associated with the progress of CVD (7).

Early studies have shown that PPARα protects the health of the cardiovascular system. Fenofibrate-induced PPARα activation protects against endothelin-induced cardiac hypertrophy and failure through negative regulation of AP-1 binding activity (103). PPARα agonists decrease macrophage-laden atherosclerotic lesions in a non-diabetic mouse model (104). PPARα agonist GW7647 treatment of LDL receptor-null mice is shown to inhibit both atherosclerosis and the formation of macrophage foam cells in the peritoneal cavity (105). Fibrate therapy results in an increase in apoA transcription and a subsequent increase in HDL levels (106); in addition, fibrates influence reverse cholesterol transport *via* an upregulation of the ATP-binding cassette transporter (ABCA1) (107) and by an increase in the hepatic uptake of HDL (108), together alleviating the progress of atherosclerosis, the main inducement of CVD.

**Figure 4:**
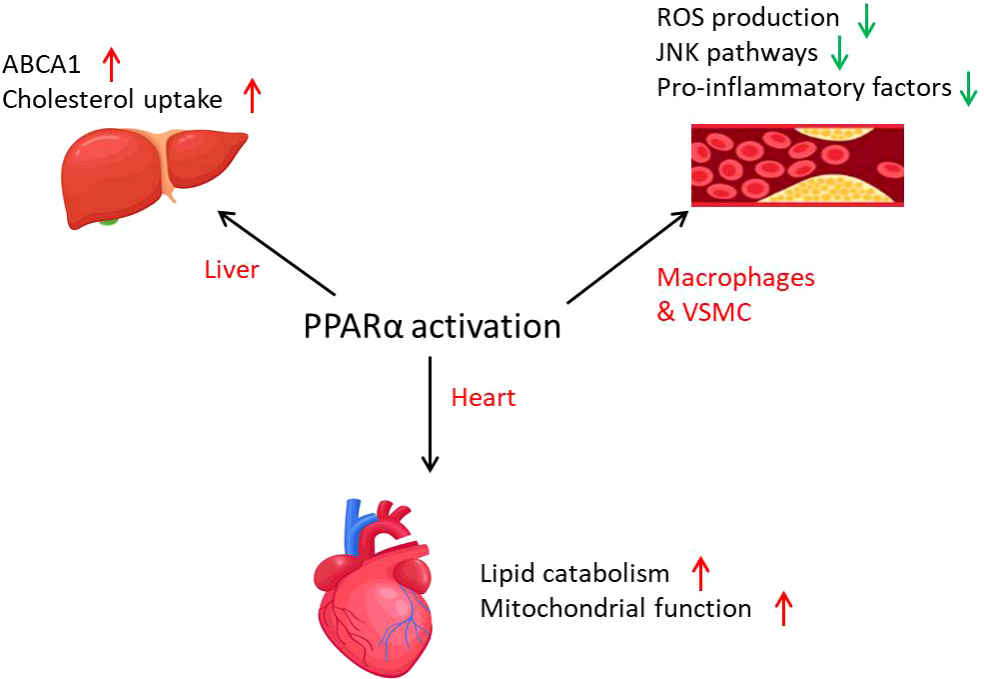
The role of PPARα in cardiovascular diseases. PPARα is involved in cardiovascular diseases through lipid metabolism activation and inflammation inhibition. PPARα activation promotes mitochondrial function and lipid catabolism in the heart and cholesterol uptake in the liver. In atherosclerosis, PPARα activation leads to the decrease of inflammation in macrophages and VSMCs.

PPARα influences CVD, which might be mainly through metabolic regulation (**Figure 4**). Cardiac-specific overexpression of PPARα results in hypertrophy and failure in association with intracellular accumulation of neutral lipids (109). Treatment of human macrophages with PPARα agonists increases the expression of cholesterol efflux proteins such as ABCA1 and SR-B1 (107). In atherosclerosis, PPARα plays important roles in lipid homeostasis in different tissues as discussed above. The mitochondrial states regulated by PPARα also influence health of the heart. Mitochondrial fatty acid oxidation causes alterations such as heart failure, ischemic heart disease, and diabetic cardiomyopathy, when the expression of PPARα is decreased (110). ATGL-mediated fat catabolism regulates cardiac mitochondrial function *via* PPARα and PGC1, where PPARα agonists completely reverse the mitochondrial defects and restore normal heart function (111). Moreover, overexpression of PPARα ameliorates doxorubicin-induced cardiotoxicity by reducing mitochondria-dependent apoptosis (112). PPARα has also been implicated in the regulation of redox responses in the endothelium, for example inducing the expression of SOD1 and also attenuating the induction of NOX in primary ECs (113, 114).

In humans, PPARα trans-repression occurs not only in the liver but also in isolated vascular endothelial cells, linking PPARα to systemic inflammation and atherosclerosis. In LDLR-deficient mice, macrophage-specific overexpression of PPARα is reported to reduce atherosclerosis (115). An *in vitro* study suggested that PPARα activation protects against cardiac hypertrophy and failure partly *via* inhibition of the JNK pathway (103). Several PPARα agonists could inhibit the synthesis of proinflammatory mediators such as IL-1-mediated activation of IL-6 and prostaglandin along with cyclooxygenase-2 through suppression of NF-κB signaling in VSMCs (116, 117). Ligand activation of PPAR-α in macrophages inhibits the activation of inducible NOS and production of TNF-α and MMP9 (7). These results investigate that PPARα regulates CVD by its metabolic and immune regulatory function, where PPARα agonists show potential in CVD treatment.

## PPARα in cancers: dual characters

**Figure 5:**
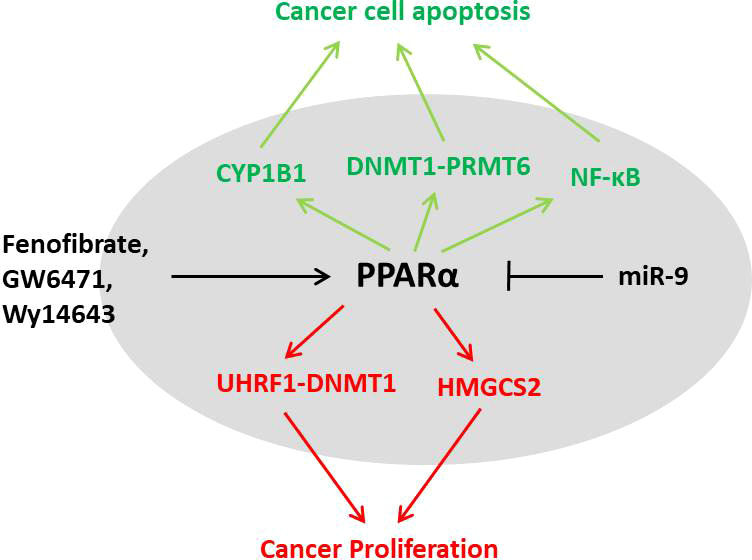
The dual characters of PPARα in cancer. PPARα activation could result in proliferation of cancer cells through the UHRF1–DNMT1 pathway and HMGCS2; however, the agonists could also lead to cell apoptosis by CYP1B, PRMT6, or NF-κB pathways. The role of PPARα in different cancers needs further investigation.

The complex roles of PPARα in cancers have recently been focused by scientists **Figure 5**. TCGA data suggest that tumor microenvironment characteristics were correlated with the expression level of PPARα in pan-cancer (118). As a highly expressed transcriptional factor for lipid metabolism in the liver, PPARα is essential for MAFLD progress. However, some studies suggest that long-term activation of PPARα induced hepatocellular carcinoma in mice and was essential for the development of hepatic steatosis (119). Administration of fibrates and Wy14643 promoted hepatocyte proliferation and resulted in significant hepatomegaly *in vivo* (120). A recent study suggests that PPARα activation promotes hepatocyte proliferation through UHRF1–CDH1-mediated epigenetic modulation (121), which might partly explain the phenotype. However, it still remains a question that PPARα is negatively correlated with HCC in human, which seems different from data in mice (122). In breast cancer, the role of PPARα also seems bidirectional. Six PPARα polymorphisms are evaluated in association with incident breast cancer, from which rs4253760 is found associated with a nearly 100% relative increase in the risk of postmenopausal breast cancer (123). Fenofibrate induces apoptosis of triple-negative breast cancer cells *via* activation of the NF-κB pathway (124), and Wy14643 shows toxicity to breast cancer cells *via* PPARα–CYP1B1 expression (125), suggesting the therapy potential of PPARα in breast cancer. However, fibrates’ influence on proliferation of breast cancer might be dose-dependent, whereas low doses of fibrates stimulate proliferation of MCF-7 cells but high doses suppress it (126). Moreover, PPARα-selective antagonist GW6471 inhibits cell growth by inducing energy imbalance and metabolic stress (127). Studies on PPARα in colorectal cancer are relatively lacking compared with breast and liver cancers. Intestinal PPARα shows protective function against colon carcinogenesis *via* regulation of methyltransferases DNMT1 and PRMT6 (128), whereas its target HMGCS2 promotes cancer proliferation in another research (129). In a word, although PPARα is associated with the progress of pan-cancer according to omics analysis, the role of PPARα in cancer remains uncertain.

## Conclusion

As a transcription factor with fatty acids to be the natural ligands, PPARα is a key TF in lipid metabolism. Its transactivation of lipid oxidation and trans-repression of inflammation, together with abundant modification and agonists, suggests that PPARα is involved in the regulation of diverse human diseases. PPARα shows protective effects in metabolic syndromes including MAFLD and diabetes, as well as its benefit for cardiovascular health. Selective agonists such as fenofibrate and Wy14643 show great potential in treatment of these diseases. However, the function of PPARα in cancer remains a puzzle.

In perspective, there are still lots of questions that should be answered in this area. Our knowledge on modification of PPARα is deficiency, which is obviously associated with metabolic conditions of the cell. How PPARα affects diabetes particularly insulin resistance needs more evidence. Whether and how PPARα directly regulates neuroinflammatory diseases lacks focus. The role of PPARα in different cancers still needs further exploration; its contradicting function in some research indicates that the function of this factor might be tightly associated with metabolic states, or even the regulation of PPARα is only a consequence of therapies. Moreover, clinical researchers should also pay attention to the targeted tissues of PPARα agonists, which might lead to a systemic influence on adipose, liver, muscle, heart, etc.

## Author contributions

YL conceived the study and accomplished the major part of writing, YW and P-FL provided perspectives and carried out the modification and perfection. All the authors contributed to the finalization of this manuscript.
